# Massive Edema of the Lower Limbs in Patients after Spinal Cord Injury—One Picture, Different Diagnoses

**DOI:** 10.3390/ijerph18084219

**Published:** 2021-04-16

**Authors:** Magdalena Mackiewicz-Milewska, Małgorzata Cisowska-Adamiak, Katarzyna Sakwińska, Iwona Szymkuć-Bukowska, Iwona Głowacka-Mrotek

**Affiliations:** Department of Rehabilitation, Collegium Medicum in Bydgoszcz, Nicolaus Copernicus University in Toruń, 85-094 Bydgoszcz, Poland; magmami@onet.eu (M.M.-M.); k.sakwinska@cm.umk.pl (K.S.); iwona.szymkuc@cm.umk.pl (I.S.-B.); iwona.glowacka@cm.umk.pl (I.G.-M.)

**Keywords:** spinal cord injury, deep vein thrombosis, heterotopic ossification, fracture

## Abstract

Diagnosis of the cause of massive edema of the lower limbs in patients after spinal cord injury (SCI) can be difficult because of loss of pain sensation, commonly occurring in this group of patients. This paper reviews several different pathologies that can lead to lower-limb edema and the associated diagnostic difficulties. We present four cases of patients with massive edemas of lower limbs at different times after SCI undergoing treatment in the Department of Rehabilitation, University Hospital in Bydgoszcz, Poland. All patients had a lack of pain sensation in the lower limbs and significantly elevated levels of D-dimer. In two cases, deep vein thrombosis (DVT) and intramuscular hematomas (IHs) were diagnosed. IHs were probably a consequence of antithrombotic treatments implemented due to the occurrence of DVT. Heterotopic ossification (HO) was diagnosed in a third case, and, in another patient, who was hospitalized for the longest period after injury, we found humeral bone fractures. Heterotopic ossification, intramuscular haematomas, and bone fractures of the lower limb can mimic DVT. Careful observation of the edema evolution is recommended, as the onset of new symptoms may indicate a different cause of edema from that initially established.

## 1. Introduction

Patients who undergo rehabilitation after spinal cord injuries (SCIs) are among the most difficult to diagnose and treat. Difficulties arise from the severity of trauma, from complications occurring in the subacute and chronic period after the injury, and because of diagnostic ambiguities resulting from sensory disorders that commonly occur in these patients. Edema of the lower limbs frequently occurs in this group of patients. It may be caused by immobilization, as well as venous and lymphatic stasis, but it can also be the result of more serious complications such as deep vein thrombosis (DVT), heterotopic ossification (HO), intramuscular hematomas (IHs), limb fractures, or cellulite [1,2]. Due to the abnormal or disturbed feeling of pain in patients after SCI, diagnosis of edema based on clinical examination is difficult. Doppler ultrasound (DUS) examination is noninvasive, easy, and fast to perform, and it is often useful for rapid diagnosis in complications such as IH, DVT, or HO.

This study presents four cases of patients after SCI with lower-limb edema of sudden onset as an outcome from different causes. To our knowledge this is the first paper that examines several different pathologies that lead to edema, illustrating the diagnostic difficulties associated with each. All patients in this study were admitted to the Department of Rehabilitation, University Hospital, Nr. 1, in Bydgoszcz, Poland. The basic characteristics of all cases is presented in Table 1.

**Case No. 1**

A 57-year-old patient was admitted 1 month after SCI at the TH12 level. Upon physical examination, flaccid paraplegia and loss of superficial sensation from the L2 level were found. Muscle strength assessed using the MRC scale was as follows: 5 in MRC in all muscle groups in upper limbs; 0 in all muscle groups of lower limbs. On the third day of hospitalization, the patient had a DUS screening examination for DVT. As a result of the DUS, DVT in the right and left knee and right femoral veins was diagnosed. At the time of diagnosis, the patient was asymptomatic with no edema, pain, or redness of lower limbs; however, D-dimer values were elevated at 8035 mg/L. The patient was given a therapeutic dose of enoxaparine. On the 11th day of low-molecular-weight heparin (LMWH) treatment, massive edema of the right lower limb was noted. A DUS performed at the onset of edema revealed a massive IH within the iliac muscle, extraperitoneally, and an IH in the rectus femoris and the vastus intermedius muscles (sized 120 mm × 80 mm × 50 mm). A CT examination of the abdomen and pelvis was performed, and a hematoma of the iliac and thigh muscles was confirmed. In the contrast study, active bleeding in the deep thigh basin was revealed. LMWH treatment was discontinued, and a vena cava filter was implanted. In subsequent days, the edema was observed to increase in both lower limbs. In the control DUS, thrombotic changes were found to be increased in both the deep veins of the thigh and the distal veins of the right and left calves. After consultation with a vascular surgeon, treatment with therapeutic doses of LMWH was resumed. Resorption of the hematoma of the right thigh was observed by DUS.

The presented case shows that the clinical course of DVT and the consequences of its treatment can be very complicated. Clinical observation of edema of the lower limbs must be daily and very accurate. Every change of the clinical picture requires the extension of laboratory diagnostics, as well as further imaging studies. As the above case shows, treatment decisions are often very difficult and related to the possibility of further complications.

**Case No. 2**

A 44-year-old patient was admitted 1 month after resection of an intramedullary tumor (schwannoma) at TH10. In the physical examination on admission, flaccid paraplegia, loss of superficial sensation from the TH10 level, and edema in both lower legs were observed. Muscle strength assessed using the MRC scale was as follows: 5 in MRC in all muscle groups in upper limbs; 0 in all muscle groups of lower limbs. A DUS of both lower limbs was performed, and DVT in both calves was found. The D-dimer value was 8159 ng/mL at admission. Treatment with therapeutic doses of LMWH was initiated, followed by oral anticoagulants. After 2 months of hospitalization, there was massive swelling of the right thigh and the DUS exam was performed again. There were no new thrombotic changes in the proximal and distal deep veins of the right lower limb; however, three IHs were found within the posterior thigh muscles with sizes of 48 mm × 39 mm, 53 mm × 47 mm, and 35 mm × 39 mm. In subsequent DUS exams, resorption of the hematomas was monitored, and their reduction was observed.

**Case No. 3**

**Figure 1:**
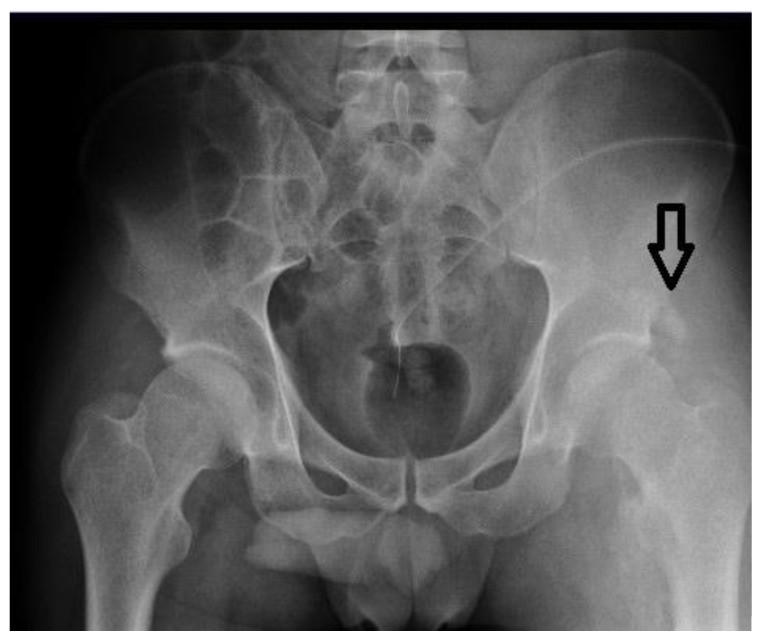
Case 3, heterotopic ossification of left hip.

A patient, age 28, with SCI at the C5–C7 level, was admitted 2.5 months after the injury. The physical examination on admission revealed paraplegia and paresis of upper limbs, elimination of superficial sensation from level C7, and massive edema of the left lower limb. Muscle strength assessed using the MRC scale was as follows: 5 in elbow flexors on each side; 2 in elbow extensors on each side; 3 in both wrist extensors, 2 in both wrist flexors; 0 in both fingers flexors; 0 in both abductors pollicis longus. The lower-limb strength was 0 on the MRC scale in all muscle groups. In laboratory tests, elevated D-dimer values up to 3534 ng/mL were observed. A DUS exam of the left lower limb did not show thrombotic changes in the superficial and deep veins. However, a massive hyperechoic signal was observed around the left hip joint, suggesting the presence of HO in the area of the joint. A pelvic X-ray revealed extensive periarticular ossification of the left hip joint (Figure 1). Active ossification was confirmed by an elevated level of alkaline phosphatase of 262 ng/mL (normal: <150 ng/mL). It should be noted that there were no pains characteristic of the active process of ossification due to sensory disorders below the SCI damage. Limitation of passive mobility of the left hip joint was also observed.

**Case No. 4**

**Figure 2:**
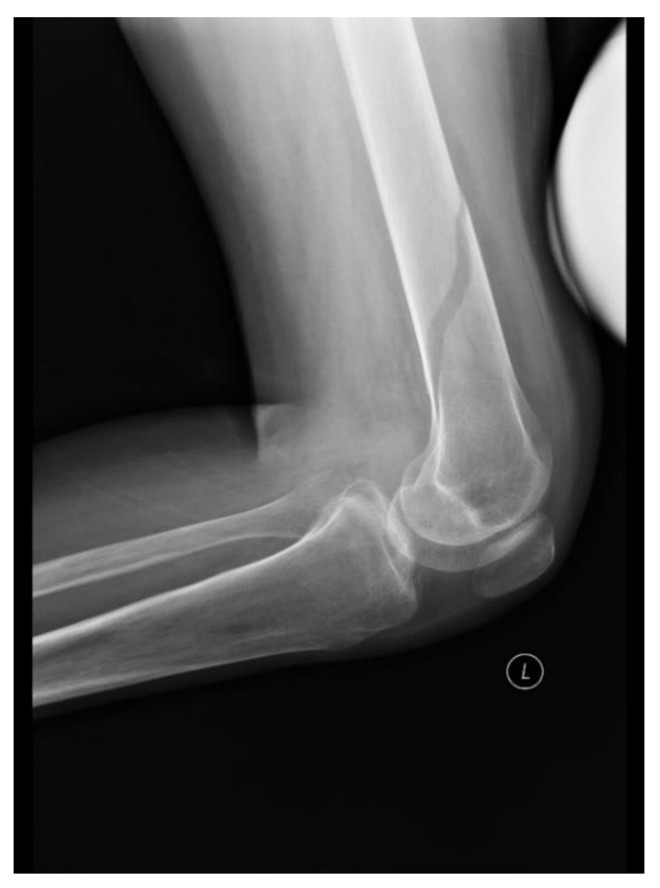
Case 4, fracture of left femoral bone.

A 30-year-old patient was admitted 56 months after SCI at the cervical level, C5. The physical examination on admission showed paraplegia and paresis of the upper limbs and abolition of superficial sensation from level C7. Muscle strength assessed using the MRC scale was as follows: 4 elbow flexors on each side; 2 in elbow extensors on each side; 4 in both wrist extensors; 3 in both wrist flexors; 2 in both fingers flexors; 0 in both abductors pollicis longus. The lower-limb strength was 0 on the MRC scale in all muscle groups. On the 14th day of hospitalization, edema of the left lower limb occurred a few hours after stretching exercises and increased throughout the next day, but the patient did not feel any pain in the limb. It was determined, on the basis of interviews with the patient and medical staff, that the patient was not injured. The third day after the edema began, a lower-limb DUS was performed, and DVT was excluded. After X-ray, a spiral fracture was found at half the height of the left femur (Figure 2). The D-dimer level on the second day after the onset of edema was 2900 ng/mL. The patient was provided with a splint.

## 2. Discussion

The primary medical condition to consider when sudden lower-limb swelling occurs in a patient after SCI is DVT. However, in this study, we would like to point out that DVT in patients after SCI does not always have to manifest as lower-limb edema (see case 1), while lower-limb edema may be a symptom of serious diseases other than DVT. The diagnosis and treatment of edema in patients after SCI is difficult, as the exacerbation of edema may result from a pathology different from that diagnosed at the beginning.

In patients 1 and 2, despite the previously diagnosed DVT, the occurrence of edema turned out to be due to a different condition, i.e., intramuscular hematomas, and, in the first case, the increase in edema resulted from exacerbation of thrombotic changes. Therefore, we would like to emphasize that, at every stage of the treatment, one must be vigilant, because the edema may change its etiology. In one patient, several causes of edema may occur in a short period of time, which require radically different treatment approaches. Lower-limb DVT occurs most often in the acute period up to the third month after injury, although it can also be observed in the chronic period [1,3,4,5]. The most important laboratory test used in the diagnosis of DVT is the D-dimer test, which is 87–100% sensitive but not very specific (approximately 55%) [6,7,8,9]. An increase in D-dimer level may occur in the course of many other pathologies, such as infections, injuries, after surgery, in neoplastic disease, and bleeding [10].

In patients with SCI, an additional difficulty in the diagnosis of DVT is a persistently high level of D-dimer in patients without DVT, both in the acute phase and in the chronic phase after injury and surgery [3,9]. In the cases presented here, all patients had D-dimer values significantly elevated above normal (<500 ng/mL). This was true for those with diagnosed thrombosis or hematoma, as well as in patients with HO or a femur fracture. Therefore, the level of D-dimer cannot be the only basis for a diagnosis of thrombosis. In similar cases, the diagnosis should be extended, with particular emphasis on ultrasound. DUS can confirm or exclude the occurrence of thrombosis, as well as visualize the presence of intramuscular hematomas, which, as our experience shows, may be a complication of anticoagulant treatment in patients after SCI [11,12]. The early stage of IH on ultrasound images as a typical anechoic space. Ultrasound examination is also good choice for monitoring hematoma resorption [13]. However, IHs are not common complications during rehabilitation of patients after SCI. The formation of a hematoma may be associated with injury to the muscles during exercises or a complication of anticoagulant use [14]. The muscles in patients after SCI may be atrophied and change depending on fiber type [15]. Yeung [16] described three cases of huge IHs in patients after SCI. All patients had anticoagulation therapy at a therapeutic dose with i.v. heparin or enoxaparin. In each case, it was necessary to reduce the heparin dose to a prophylactic level. In two cases, the hematomas required incision and drainage and, in one case, a filter was implantated into the inferior vena cava. Similarly, in our patient 1, the large hematoma required a dose reduction of heparin, which was associated with increased DVT, followed by filter implantation.

Case 3 presents a patient whose edema was the result of HO formation. The clinical picture also mimicked DVT, especially with the significant increase in D-dimer to 3534 ng/mL. Neurogenic heterotopic ossifications (NHOs) can form most often after brain injuries (11–73%), after spinal cord injuries (10–53%), or as a result of anoxic brain injuries [17,18,19].

Radiological diagnosis of HO is established mainly by X-ray or CT. Nevertheless, ultrasound examination is even more often used in the detection of ossifications due to its high sensitivity [20,21,22]. Detecting HO in patients with SCI can be difficult because of the impaired or absent pain sensation, as joint pain and limited range of motion are the main symptoms of ossification. Additional tests that may support a diagnosis of ossification include an increased level of alkaline phosphatase, which was elevated in our patient at 262 ng/mL (normal: <150 ng/mL). A similar case was described by Scola, in which left lower-limb edema appeared in a patient after multiorgan trauma, including SCI with paraplegia. Initially DVT was susepcted; however, during ultrasound examination after excluding thrombosis, calcifications in the area of the vein were found in the soft tissues of the thigh. Further diagnostics confirmed the presence of neurogenic ossification in the hip joint. It should also be noted that both HO and DVT appear mainly in the early period after the injury, up to 3 months [22].

A case of HO imitating DVT was also described by Bang [1] in a 35 year old patient with tetraplegia after SCI, who developed edema 1 month after the injury and had redness and increased calf temperature, normal D-dimer levels, and slightly elevated APL at 140 U/L (norm to 129 U/L). A DUS was performed, which excluded DVT, and computed tomography venography (CTV) visualized HO in the anteromedial part of the thigh, which was compressing the veins. Although the D-dimer level in the reported case was normal, in our patient, it was significantly elevated at 3534 ng/mL, which made diagnosis more difficult. In our opinion, compression of the veins by HO or hematoma may be an additional risk factor for the development of DVT in SCI patients; therefore, they should be monitored by ultrasound examination, especially until the edema subsides, in order to exclude the appearance of DVT as a complication.

The fourth case of sudden edema was due to a bone fracture. The initially suspected DVT was not confirmed by DUS examination, and the diagnosis was made difficult by the loss of pain sensation and the absence of trauma in the history. Fracture is a common consequence of osteoporosis, which is a known complication in SCI patients [23]. The number of fractures increases over time from injury from 1% in the first year after injury to 4.6% at 20 years after injury [23,24]. Fractures are mostly caused by minimal trauma, and the most common causes are transfers or changing position in bed [25]. In our case, the probable cause established post factum was the stretching exercises. Our clinical experience has shown that even correctly conducted physiotherapy may lead, in rare cases, to fractures, especially in patients after SCI with increased muscle tension.

## 3. Conclusions

Sudden limb edema in patients after SCI requires immediate testing for DVT as a serious complication that may lead to pulmonary embolism. If DUS examination shows absence of DVT, then further diagnosis is required. DUS is a simple, low-cost, repeatable tool that is the basis of diagnosis of acute limb edema in patients with SCI. However, if DVT or hematoma is not confirmed, CT or X-ray diagnostics should be performed to check for HO or fractures. Heterotopic ossification, intramuscular hematoma, or bone fractures of the lower limb can mimic DVT. Careful observation of the evolution of edema is recommended, as the onset of new symptoms may indicate a different cause of edema from what was previously established. In the described cases, high D-dimer levels turned out to be a sensitive test in the diagnosis of DVT, but not very specific. In the post-trauma period, one must always consider the presence of a lower-limb fracture, even if there has been no trauma. Ultrasonography is highly recommended for monitoring the treatment process, especially in cases of intramuscular hematomas.

## Figures and Tables

**Table 1:** Characteristics of presented cases.

Case	Age	Time from Injury	Level of Injury	Type of Paresis	Muscle Tension	D-Dimer Level (mg/L)	Cause of Edema
1	57	1 month	Th12	Paraplegia	Flaccid	8035	IH DVT
2	44	1 month	Th10	Paraplegia	Flaccid	8159	DVT IH
3	28	2.5 months	C5–C7	Paresis of upper limbs, paraplegia	Flaccid	3534	HO
4	30	63 months	C5	Paresis of upper limbs, paraplegia	Spastic (MAS 3/4)	2900	Fracture

Abbreviations: MAS—modified Ashworth scale, DVT—deep vein thrombosis, IHs—intramuscular hematomas, HO—heterotopic ossification.

## Data Availability

The data presented in this study are available on request from the corresponding author. The data are not publicly available due to privacy.
